# Different epidemiological profiles in patients with Zika and dengue infection in Tapachula, Chiapas in Mexico (2016–2018): an observational, prospective cohort study

**DOI:** 10.1186/s12879-021-06520-x

**Published:** 2021-08-28

**Authors:** Pablo F. Belaunzarán-Zamudio, Héctor Armando Rincón León, Sandra Caballero Sosa, Emilia Ruiz, José Gabriel Nájera Cancino, Paul Rodriguez de La Rosa, María de Lourdes Guerrero Almeida, John H. Powers, John H. Beigel, Sally Hunsberger, Karina Trujillo, Pilar Ramos, Fernando J. Arteaga-Cabello, Alexander López-Roblero, Raydel Valdés-Salgado, Hugo Arroyo-Figueroa, Eli Becerril, Guillermo Ruiz-Palacios, for the Mexican Emerging Infectious Diseases Clinical Research Network (La Red), Justino Regalado Pineda, Héctor Armando Rincón-León, Karla R. Navarro-Fuentes, Sandra Caballero-Sosa, Francisco Camas-Durán, Zoyla Priego-Smith, Emilia Ruiz, José Gabriel Nájera-Cancino, Paul Rodriguez De la Rosa, Jesús Sepúlveda-Delgado, Alfredo Vera Maloof, Karina Trujillo, Alexander López-Roblero, Raydel Valdés-Salgado, Yolanda Bertucci, Isabel Trejos, Luis Diego Villalobos, Pablo F. Belaunzarán-Zamudio, Pilar Ramos, Fernando J. Arteaga-Cabello, Lourdes Guerrero, Guillermo Ruiz-Palacios, Paola del Carmen Guerra Blas, Luis Mendoza-Garcés, Samira Toledo Roy, Hugo Arroyo-Figueroa, Peter Quidgley, Laura Macedo, Eli Becerril, Abelardo Montenegro Liendo, John H. Powers, John H. Beigel, Sally Hunsberger

**Affiliations:** 1 Departamento de Infectología, Instituto Nacional de Ciencias Médicas y Nutrición Salvador Zubirán, Mexico City, Mexico; 2 National Institute of Allergy and Infectious Diseases, Bethesda, MD USA; 3 Unidad de Medicina Familiar No.11, Instituto Mexicano del Seguro Social, Tapachula, Chiapas Mexico; 4 Clínica Hospital Dr. Roberto Nettel Flores, Instituto de Seguridad y Servicios Sociales de los Trabajadores del Estado, Tapachula, Chiapas Mexico; 5 Hospital General de Tapachula, Tapachula, Chiapas Mexico; 6 Hospital Regional de Alta Especialidad Ciudad Salud, Tapachula, Chiapas Mexico; 7 Leidos Biomedical Research, Inc., Frederick National Laboratory for Cancer Research, Frederick, MD USA; 8 Westat, Rockville, MD USA; 9 Mexican Emerging Infectious Diseases Clinical Research Network (La Red), Mexico City, Mexico

**Keywords:** Zika, Dengue, Chikungunya, Emerging diseases, Outbreak, Mexico

## Abstract

**Background:**

The introduction of Zika and chikungunya to dengue hyperendemic regions increased interest in better understanding characteristics of these infections. We conducted a cohort study in Mexico to evaluate the natural history of Zika infection. We describe here the frequency of Zika, chikungunya and dengue virus infections immediately after Zika introduction in Mexico, and baseline characteristics of participants for each type of infection.

**Methods:**

Prospective, observational cohort evaluating the natural history of Zika virus infection in the Mexico-Guatemala border area. Patients with fever, rash or both, meeting the modified criteria of PAHO for probable Zika cases were enrolled (June 2016–July 2018) and followed-up for 6 months. We collected data on sociodemographic, environmental exposure, clinical and laboratory characteristics. Diagnosis was established based on viral RNA identification in serum and urine samples using RT-PCR for Zika, chikungunya, and dengue. We describe the baseline sociodemographic and environmental exposure characteristics of participants according to diagnosis, and the frequency of these infections over a two-year period immediately after Zika introduction in Mexico.

**Results:**

We enrolled 427 participants. Most patients (n = 307, 65.7%) had an acute illness episode with no identified pathogen (UIE), 37 (8%) Zika, 82 (17.6%) dengue, and 1 (0.2%) chikungunya. In 2016 Zika predominated, declined in 2017 and disappeared in 2018; while dengue increased after 2017. Patients with dengue were more likely to be men, younger, and with lower education than those with Zika and UIE. They also reported closer contact with water sources, and with other people diagnosed with dengue. Participants with Zika reported sexual exposure more frequently than people with dengue and UIE. Zika was more likely to be identified in urine while dengue was more likely found in blood in the first seven days of symptoms; but PCR results for both were similar at day 7–14 after symptom onset.

**Conclusions:**

During the first 2 years of Zika introduction to this dengue hyper-endemic region, frequency of Zika peaked and fell over a two-year period; while dengue progressively increased with a predominance in 2018. Different epidemiologic patterns between Zika, dengue and UIE were observed. *Trial registration* Clinical.Trials.gov (NCT02831699).

**Supplementary Information:**

The online version contains supplementary material available at 10.1186/s12879-021-06520-x.

## Background

Forty-five years after the re-emergence of dengue in the Americas, the region saw the introduction of two vector-transmitted viruses with Zika and chikungunya in 2014 followed by their subsequent rapid expansion. While the Zika outbreak approached pandemic proportions in 2015 [1–3], phylogenetic and molecular diagnostics studies identified the introduction of Zika as early as 2012–2013 [4, 5]. Similar to dengue, chikungunya and Zika viruses have become endemic in Latin America along with West Nile virus, Venezuelan equine encephalitis and other arboviruses [6, 7]. It seems likely that chikungunya and Zika will continue to circulate at lower levels following the initial outbreaks. The introduction of these viruses, in addition to previously endemic circulating members of the family *Flaviviridae*, and co-circulating bacterial, parasitic and viral pathogens causing similar disease presentations, has raised the interest in better understanding clinical and epidemiological characteristics of these infections [3, 8].

This study (Zik01) was launched in March 2016, in response to the Zika outbreak. Our motivation was to study the natural history of Zika and the short- and long-term complications of this disease, with emphasis on the neurological complications, and compare these to other similar diseases including, dengue and chikungunya. We also aimed to estimate the proportion of participants with fever, rash or both who had a confirmed diagnosis of Zika, chikungunya or dengue or co-infections with these viruses in Tapachula, Chiapas, Mexico. In this manuscript, we describe the overall study design and procedures of the Zik01 study, which encompasses the febrile-rash, asymptomatic household, Guillain–Barre and pregnant cohorts. In addition, we describe a limited set of baseline clinical and epidemiologic results according to confirmed diagnosis from the febrile-rash cohort, and the asymptomatic household cohort. We also describe frequency trends over a 2-year period immediately after Zika introduction in Mexico.

## Methods

### Study design, settings and study population

**Map 1:**
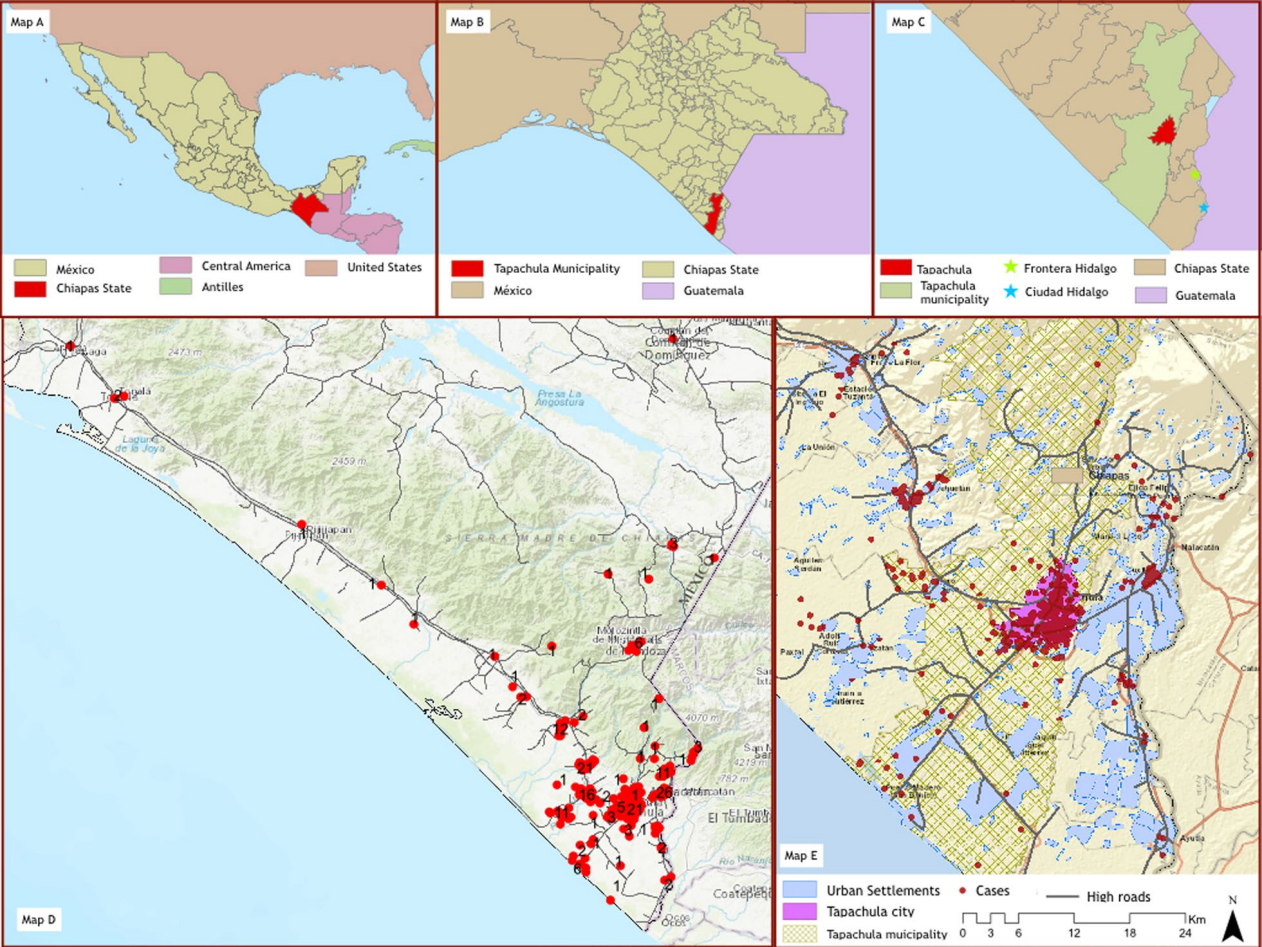
Location of **A** the State of Chiapas, Mexico in **B** the border with Guatemala, where **C** the city of Tapachula is located. Participants were enrolled in 4 participating health care centers and lived in the urban area of Tapachula and 14 rural municipalities in its periphery (**C**). The red dots in maps **C** and **D** indicate the neigborhood or communities of residence of participants. The numbers in black in map C indicate the quantity of participants living in the community enrolled in the study. Each red dot in map E represent an individual participant distributed in the communities around the city of Tapachula. Map developed by Taller de Analisis Espacial (http://taearquitectos.com.mx/) using OpenStreetMap ( https://www.openstreetmap.org/#map=5/38.007/-95.844) and QGIS 3.2 (https://qgis.org/en/site/about/index.html) themed with own data. QGIS is a free and open-source General Public License (GNU) Geografic Information System (GIS). OpenStreetMap (OSM) is a free Open Database Licence (ODbl) editable map of the world

This was a prospective, observational, longitudinal, cohort study. The study was conducted in the city of Tapachula, Chiapas, located 23 km west of the border with Guatemala along the Pacific coast (see Map 1). This area is considered hyperendemic for dengue, with an estimated seroprevalence of 83% (95% CI 73.8–88.9) in school children aged 13–17 years [9]. Participants were enrolled in two primary healthcare centers from Instituto Mexicano del Seguro Social—Mexican Institute of Social Security (Unidad de Medicina Familiar No.11), and from Instituto de Seguridad y Servicios Sociales de los Trabajadores del Estado -Institute of Social Security and Services for Government Employees (Clínica Dr. Roberto Nettel Flores), one General Hospital from the State of Chiapas Ministry of Health (Hospital General de Tapachula) and a tertiary care hospital from the National Ministry of Health (Hospital Regional de Alta Especialidad Ciudad Salud). The cohort is coordinated from the Mexican Emerging Infectious Disease Clinical Research Network (La Red) (https://www.redmexei.mx/) at the Instituto Nacional de Ciencias Médicas y Nutrición Salvador Zubirán in Mexico City and supported by the National Institute of Allergy and Infectious Diseases, National Institutes of Health in Bethesda, MD, USA.

**Fig. 1:**
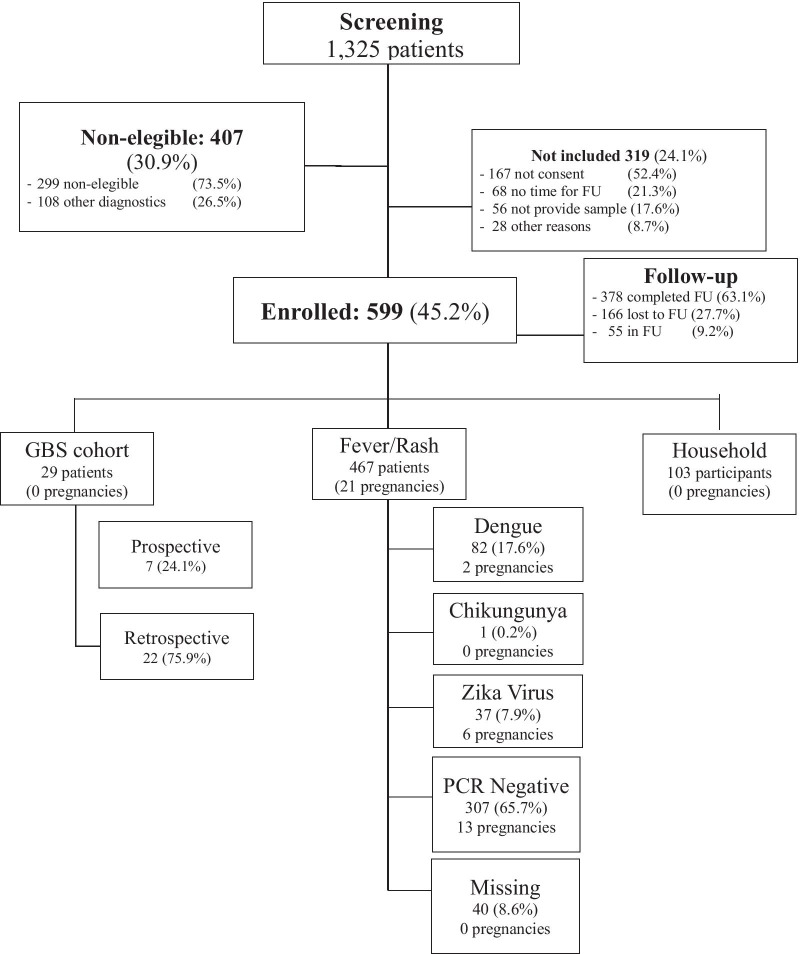
Screening and enrollment of patients with symptoms compatible with Zika infection in Tapachula, Chiapas (cohort Zik01. Mexico, 2016–2018). Description: Flow diagram showing screening and enrollment of study population 1 There were 40 (8.6%) participants on whom we did not have enough samples to rule out any of these infections (absence of Zika, chikungunya and dengue in available samples but missing data for at least two time points)

We enrolled participants of all ages in two parallel cohorts: a febrile-rash cohort (attempting to capture acute Zika/chikungunya/dengue infection) and a household cohort (attempting to capture asymptomatic or minimally symptomatic participants in contact with persons in the febrile-rash cohort). Participants were enrolled in the febrile-rash cohort if they met criteria for suspected Zika virus infection. We used a modified version of the World Health Organization and the Pan American Health Organization probable case definition [10] which comprised any two of the following symptoms: rash or elevated body temperature (> 37.2 °C) accompanied with at least one of the following: arthralgia, myalgia, non-purulent conjunctivitis or conjunctival hyperemia, or headache or malaise in the 7 previous days before the initial visit, with no obvious alternative diagnosis to explain the symptoms. Asymptomatic household contacts of participants in the febrile-rash cohort, were invited to participate in the household cohort if they lived in Tapachula or nearby areas. They were contacted by phone and if they consented, a research team scheduled an appointment to visit the household to enroll all members of the household present during the visit, including minors (See Map 1). Consent to participate and information from minors were obtained through their parents. Consent and assent to participate was on an individual basis. The protocol established that members of the household cohort would be referred to the corresponding clinic to enrollment in the febrile-rash cohort if symptomatic during the household visit. There were two additional cohorts: women with febrile-rash diseases during pregnancy and patients with Guillain–Barre syndrome, that will be describe elsewhere (Fig. 1). At the time of study planning, we decided to enroll up to 600 participants across all cohorts based on convenience and feasibility, considering the uncertainties around the natural history of the infection and its frequency during pathogen introduction in the area.

### Procedures and follow-up

Participants in the febrile-rash cohort were evaluated in the clinics at baseline, and 3, 7, 28, and 180 days after enrollment. After completing the procedures for the baseline visit, participants were asked about the number and age of household members. We requested permission and contact numbers to schedule a visit to the households to accrue their contacts for the asymptomatic household cohort. Participants in the household cohort were evaluated in their home in an initial visit (baseline) and 28 days later. During the baseline visit for each participant, we collected demographic and medical and exposure histories; obtained responses to a symptom questionnaire and performed a complete physical, including an extensive neurological examination, a brief screening for neurocognitive dysfunction (Montreal Cognitive Assessment, MoCA) [11], an assessment for disability (12-item World Health Organization Disability Assessment Schedule 2 or WHODAS 12, in Spanish) [12], and complications, including hospitalizations. Blood samples for Zika, dengue and chikungunya serologies and blood and urine for viral nucleic acid detection were drawn for all participants, and complete blood count and clinical chemistry for participants in the febrile-rash cohort. During all follow-up visits we repeated the symptom questionnaire and complete physical examination, the MoCA and WHODAS tests, the assessment for complications, and draw blood samples to repeat serologies, and blood and urine samples PCR for viral nucleic acid detection in all participants in all cohorts. Patients in the febrile-rash cohort had their first sample within 7 days after symptoms onset as recommended by the CDC guidance for Zika [13]. As reported elsewhere [14], four real-time RT-PCR assays were performed simultaneously in blood and urine samples from baseline visits and 3, 7 and 28 days later, screening for Zika [15], dengue [16], chikungunya [17] and panflavivirus [13]. Briefly, for RNA extraction,total nucleic acids from 500 µl of serum and urine were extracted using the NucliSENS^®^ easyMAG^®^ system (bioMerieux^®^, Netherlands) and eluted in 55 µl, according to manufacturer instructions. The amplification of the human RNaseP (RP) gene was carried out for each sample as an internal control to demonstrate the presence of RNA and the validation of the extraction process. The amplification of the NS5 gene was also carried out for the generic detection of flaviviruses as another control of Zika and dengue and to determine the possible presence of other flaviviruses in the sample.

Amplifications were performed in singleplex (each virus detected in a separate reaction) by one-step RT-PCR reaction in 25 µl with SuperScript III Platinum One-Step quantitative RT-PCR System (Invitrogen^®^, ThermoFisher Scientific^®^, Waltham, MA, USA) and 5 µl of sample. Cycle sequencing was: retrotranscription at 50 ºC for 30 min, initial PCR denaturation at 94 ºC for 2 min followed by 45 cycles of denaturation at 94 ºC for 15 s and annealing and extension at 60 ºC for 1 min in the Applied Biosystems 7500 Fast Real Time PCR System (Applied Biosystems, ThermoFisher Scientific^®^, Waltham, MA, USA).

We measured serum IgG and IgM antibodies against the three viruses using commercial Enzyme-linked immunosorbent assay (ELISA) kits: anti-ZIKV specific-IgM (ZIKV-sIgM) and IgG (ZIKV-sIgG) ELISA (EuroImmun^®^, Lübeck, Germany), anti-CHIKV specific-IgM (CHIKV-sIgM) and IgG (CHIKV-sIgG) ELISA (EuroImmun^®^, Lübeck, Germany), anti-DENV specific-IgG (Panbio Dengue IgG Capture ELISA—panbio diagnostics^®^, Republic of Korea) and anti-DENV specific IgG (Panbio Dengue IgM Capture ELISA—panbio diagnostics^®^, Republic of Korea). Considering the potential cross-reactivity and overlapping results between Zika and dengue antibodies we used serologies exclusively to explore their utility in the differential diagnostic algorithm, but definitions of confirmed cases were based exclusively on RT-PCR tests. The results of serological analyses have been presented elsewhere [14, 18]

### Definitions

We defined confirmed cases of Zika, dengue and chikungunya if viral RNA for each virus was present in serum or urine samples at any time during follow-up [19]. The absence of Zika, chikungunya and dengue RNA in serum or urine samples at any time during follow-up was defined as an undefined illness episode (UIE). Participants on whom we did not have enough samples to rule out any of these infections (absence of Zika, chikungunya and dengue in available samples but missing data for at least two time points) were defined as probable cases with missing data and excluded from this analysis.

### Statistical analyses

We describe the characteristics of participants by type of infection using simple proportions for binary variables and medians and 25th and 75th percentiles (referred as IQR for short) for continuous variables. We used 2-side p-value from Fisher’s exact test to compare proportions and difference between continuous variables were tested by nonparametric Wilcoxon rank sum test. We describe the temporal trends of infections during the study period using an epidemic curve reporting the number of confirmed cases of Zika, dengue, chikungunya virus infections, and UIE by month. The proportion of patients with blood and urine samples with detectable RNA for each virus at each visit is described using simple proportions. We describe the sociodemographic and exposure characteristics of each group in three age-strata (< 18 year old, ≥ 18 to 64 year old and ≥ 65 year old) to analyze for potential confounding effects. All analyses were conducted using SAS software, version 9.4 (SAS Institute).

## Results

### Enrollment

Centers began enrolling participants in the cohort on June 21, 2016. The screening and enrollment process are summarized in Fig. 1, with enrollment closing on July 18, 2018. Rates of enrollment and reasons for not participating varied by center. We enrolled 467 people with fever, rash or both.

### Frequency of Zika, dengue and chikungunya virus infections

There were 37 (8%) confirmed cases of Zika, 82 (17.6%) of dengue, 1 (0.2%) of chikungunya, and 307 (65.7%) UIE. We observed no coinfections. There were 40 (8.6%) participants on whom we did not have enough samples to rule out any of these infections. We enrolled 21 pregnant women (all of them in the febrile-rash cohort and presented as part of this cohort here), most of them (62%) had PCR negative results; six were confirmed Zika cases, two were confirmed Dengue cases and one was a confirmed case of Chikungunya. We identified 29 patients with GBS during the study (seven were identified prospectively). All participants with GBS prospectively enrolled were classified in the UIE group. In the retrospective cohort sample collection was scheduled at enrollment, therefore none of them had positive PCR. In the household cohort we enrolled 103 participants linked to index cases in the febrile-rash cohort. These participants were enrolled between June 2016 and July 2018. All participants in the household cohort were related to patients in the UIE group, none met criteria for enrollment in the febrile-rash cohort, and all were rt-PCR negative for Zika, dengue and chikungunya. No samples were positive for generic flaviviruses while negative for either Zika or dengue, so no other flavivirus infections were identified. All positive samples for generic flaviviruses were also positive for either dengue or Zika.

### Characteristics of the study population

Sociodemographic characteristic of participants by group are summarized in Table 1. Overall, participants with dengue were more frequently men, with a median age 10 years younger than participants with Zika and UIE and had lower education status. They also had comorbidities less frequently, as would be expected with their younger age. Participants with Zika and UIE had a higher prevalence of hypertension, diabetes, and people with UIE self-reported more frequently osteoarthrosis and chronic peripheral neuropathies.

**Table 1:** Sociodemographic characteristics of patients with symptoms compatible with Zika^1^ in Tapachula, Chiapas, Mexico (June 2016-August 2018)

Characteristic^3^	Type of confirmed infection^2^ (n = 427)			
	Zika (n = 37)	Dengue (n = 82)	Undefined illness episode (n = 307)	Household Cohort (n = 103)
Female^4^	23 (62.2%)	43 (52.4%)	190 (61.9%)	57 (55.3%)
Age	33 (13, 59)	22.5 (6, 68)	31 (5, 76)	39 (3.91)
< 18 year old	2 (5.4%)	25 (30.5%)	42 (13.7%)	9 (8.7%)
≥ 18–65 year old	35 (94.6%)	57 (69.5%)	265 (86.3%)	91(88.3)
> 65 year old	0 (0.0%)	1 (1.2%)	9(2.9%)	3 (2.9%)
Education				
No school	2 (5.4%)	3 (3.7%)	11 (3.6%)	10 (9.7%)
Basic (Degrees 1–6)	4 (10.8%)	25 (30.5%)	44 (14.3%)	12 (11.7%)
Highschool (7–12)	13 (35.1%)	35 (42.7%)	114 (37.1%)	30 (29.1%)
College	16 (43.2%)	11 (13.4%)	104 (33.9%)	30 (29.1%)
Postgraduate	2 (5.4%)	8 (9.8%)	33 (10.7%)	2 (1.9%)
Race/ethnicity				
White	7 (18.9%)	19 (23.2%)	78 (25.4%)	6 (5.8%)
Indigenous	0 (0%)	1 (1.2%)	1 (0.3%)	0 (0.0%)
Mestizo	30 (81.1%)	62 (75.6%)	227 (73.9%)	97 (94.2%)
Location				
Tapachula	20 (54.1%)	49 (59.8%)	239 (77.9%)	72 (69.9%)
Other	17 (45.9%)	33 (40.2%)	68 (22.1%)	31 (30.1%)
Comorbidities				
Skin diseases	0 (0%)	0 (0%)	1 (0.3%)	1 (1.0%)
Hypertension	2 (5.4%)	2 (2.4%)	23 (7.5%)	15 (14.6%)
Diabetes	2 (5.4%)	0 (0%)	8 (2.6%)	13 (12.6%)
Arthritis/Osteoarthritis	0 (0%)	0 (0%)	5 (1.6%)	6 (5.8%)
Chronic peripheral neuropathy	0 (0%)	1 (1.2%)	5 (1.6%)	6 (5.8%)
Guillain–Barre Syndrome	0 (0%)	0 (0%)	1 (0.3%)	1 (1.0%)
HIV	0 (0%)	0 (0%)	1 (0.3%)	0 (0.0%)

^1^Probable Zika infection cases were defined using a modified version of the World Health Organization and the Pan American Health Organization definition [6] which comprised any two of the following symptoms: rash or elevated body temperature (> 37.2 °C) accompanied with at least one of the following symptoms: arthralgia, myalgia, non-purulent conjunctivitis or conjunctival hyperemia, or headache or malaise in the 7 previous days before the initial visit, with no obvious alternative diagnosis to explain the symptoms. ^2^Confirmed Zika and dengue infections were defined as the presence of viral RNA in serum or urine samples at any time during follow-up [14]. The absence of Zika, chikungunya and dengue RNA in serum or urine samples at any time during follow-up was defined as an undefined illness episodes (UIE). ^3^Continuous variables are summarized using medians and range. ^4^Six (26%) of the 23 women in the Zika group were pregnant, two (8%) in the dengue group and 13 (56%) in the UIE. There were no pregnancies in the household cohort

### Description of epidemiological characteristics

More than 70% of participants experienced fever, but that proportion was higher among patients with dengue (95.1%). Fewer patients with UIE developed rash (36.2%), compared to almost 60% among those with dengue or Zika (Table 2). Conjunctivitis did not differ across groups. Overall, patients with dengue waited longer before seeking medical care (median: 5 days) than patient with Zika (median 3 days) and UIE (median 4 days). Patients with fever and dengue tended to seek care later after symptom onset (5 days) than dengue patients presenting with conjunctivitis (4 days) and rash (3 days), unlike patients with Zika and UIE, where the period between symptoms onset and first visit is similar for fever, rash and conjunctivitis (Table 2).

**Table 2:** Baseline characteristics of patients seeking care due symptoms compatible with Zika in Tapachula, Chiapas (Mexico, 2015–2018) (N = 427)

Clinical manifestation	Zika (n = 37)	Dengue (n = 82)	Unidentified illness episode (n = 307)
Days between first symptom and visit	3 (0–6) *	5 (1–7)*^,≠^	4 (0–7)^≠^
< 18 year old	4 (3–5)	5 (1–7)	4 (1–7)
18–64 year old	3 (0–6)	5.0 (1–7)	4.0 (0–7)
> 65 year old	–	5.0 (5–5)	4.0 (1–6)
Fever, (> 37.2 °C)^1^	26 (70.3%)*	78 (95.1%)*^,≠^	258 (84%)^≠^
< 18 year old	2 (100.0%)	25 (100.0%)	37 (88.1%)
18–64 year old	23 (65.7%)	52 (92.9%)	212 (82.8%)
> 65 year old	–	1 (100.0%)	9 (100.0%)
Days between onset of fever and visit	3 (0–6)*^,#^	5 (1–7) *^,≠^	3 (0–7)^≠,#^
< 18 year old	2.5 (2–3)	5.0 (1–7)	4.0 (0–7)
18–64 year old	3.0(0–6)	4.5 (1–7)	3.0 (0–7)
> 65 year old	–	5.0 (5–5)	4.0 (1–6)
Conjunctivitis^1^	17 (45.9%)	30 (36.6%)	126 (41%)
< 18 year old	0 (0.0%)	12 (48.0%)	14 (33.3%)
18–64 year old	16 (45.7%)	17 (30.4%)	104 (40.6%)
> 65 year old	–	0 (0.0%)	6 (66.7%)
Days between onset of conjunctivitis and visit	2 (0–5)*^,#^	4 (1–7)*^,≠^	3 (0–7)^≠,#^
< 18 year old	–	3.5 (1–7)	3.0 (1–6)
18–64 year old	2.0 (0–5)	5.0 (2–7)	3.0 (0–7)
> 65 year old	–	–	4.5(1–6)
Rash^1^	22 (59.5%)^#^	48 (58.5%)^≠^	111 (36.2%)^≠,#^
< 18 year old	0 (0.0%)	17 (68.0%)	17 (40.5%)
18-64 year old	21 (60.0%)	29 (51.8%)	90 (35.2%)
> 65 year old		1 (100.0%)	4 (44.4%)
Days between onset of rash and visit	2 (0–6)	3 (0–7)^≠^	2 (0–7)^≠^
< 18 year old	–	3.0 (1–7)	2.0 (0–6)
18–64 year old	2.0 (0–6)	2.0 (0–7)	2.0(0–7)
> 65 year old		5.0 (5–5)	1.5 (1–3)

^1^One or more of these were part of entry criteria. *p-value < 0.005 for the comparison of Zika and Dengue; ^#^*p*-value < 0.05 for the comparison of ZikV and UIE ≠ *p*-value < 0.05 for the comparison of Dengue and UIE. Comparisons with Fisher’s exact test for categorical variables and Wilcoxon rank sum test for continuous

The characteristics of the type of environmental exposures are summarized in Table 3. A higher proportion of participants with Zika self-reported having had sex with an ill partner with fever, rash or other acute illness in the previous 2 weeks of presenting symptoms than patients with dengue or UIE. This group also reported less use of screens to keep out mosquitoes at home. In comparison, participants with dengue, reported having a family member or a neighbor recently diagnosed with dengue more frequently than Zika patients having a recent contact with another person with Zika or dengue. Also, participants with dengue more frequently lived within 1 km to a standing water source than their counterparts with Zika or UIE. We performed an age-stratified analysis but observed no differences (See Additional file 1), so we present the overall distribution of exposures in Table 3.

**Table 3:** Description of exposure to arbovirus in patients with symptoms compatible with Zika infection in Tapachula, Chiapas (Mexico, 2015–2018)

Exposure^1^		Zika (n = 37)	Dengue (n = 82)	Undefined illness episode (n = 307)	Household cohort (n = 103)
Sexual relations with anyone who had a rash, fever, or other acute illness in the previous 15 days to symptom initiation		8 (21.6%)	5 (6.1%)	35 (11.4%)	10 (9.7%)
A family member of the participant was diagnosed in the last 15 days with some of the following:	Zika	1 (2.7%)	0 (0%)	2 (0.7%)	18 (17.5%)
	Dengue	1 (2.7%)	5 (6.1%)	5 (1.6%)	42 (40.8%)
	Chikungunya	1 (2.7%)	0 (0%)	1 (0.3%)	10 (9.7%)
A coworker or classmate (for children) of the participant was diagnosed in the last 15 days with some of the following	Zika	1 (2.7%)	1 (1.2%)	2 (0.7%)	0 (0.0%)
	Dengue	1 (2.7%)	1 (1.2%)	5 (1.6%)	3 (2.9%)
	Chikungunya	0 (0%)	1 (1.2%)	4 (1.3%)	1 (3.4%)
A neighbor of the participant was diagnosed in the last 15 days with some of the following:	Zika	0 (0%)	2 (2.4%)	3 (1%)	2 (1.9%)
	Dengue	1 (2.7%)	16 (19.5%)	12 (3.9%)	8 (7.8%)
	Chikungunya	0 (0%)	1 (1.2%)	3 (1%)	3 (2.9%)
Participant’s house is located within 1 km to a standing water source		16 (43.2%)	65 (79.3%)	180 (58.6%)	66 (64.1%)
Participant’s houses do not have screens to keep out the mosquitoes		9 (24.3%)	8 (9.8%)	51 (16.6%)	11 (10.7%)
At participant’s home beds and/or cribs are not covered with mosquito nets		11 (29.7%)	38 (46.3%)	55 (17.9%)	37 (35.9%)

^1^Self-reported at baseline

**Fig. 2:**
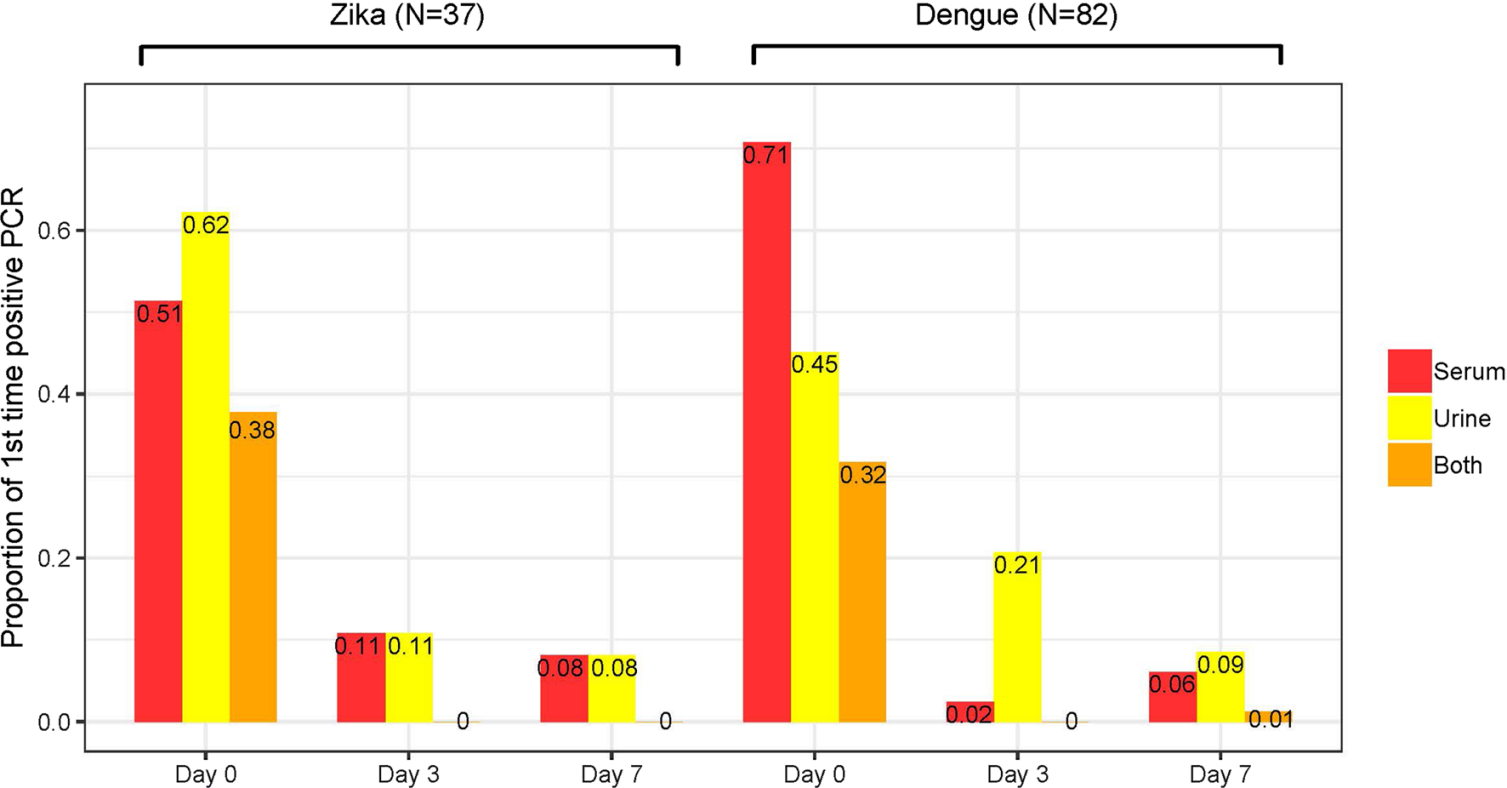
Proportion of urine and serum samples that tested positive for dengue and Zika viral RNA at baseline (day 0–7 of symptom onset), and follow-up visits at day 3 (days 8–10 of symptoms onset) and 7 (days 7–14 of symptoms onset) after enrollment in (cohort Zik01. Mexico, 2016–2018). Description: Bars figure showing the proportion of patients that tested positive for Zika and dengue at visits on day 0, 3 and 7 after enrollment in the cohort. All samples were tested for dengue and Zika viral RNA at days 14, 28 and 180 days after enrollment but none tested positive. There were no patients with dual infection

Figure 2 presents the frequency of viral RNA identification in urine and plasma samples at enrollment (Day 1–7 of symptom onset) and during follow-up for participants with confirmed Zika and dengue infection. We present only data at Days 3 and 7 of follow-up, since all samples from visits at Days 28 and 180 tested negative. At enrollment Zika was more likely to be identified in urine (62%) than dengue (45%); and dengue was more likely to be identified in plasma (71%) than Zika (51%). The frequency of viral RNA in plasma decreased faster in participants with dengue than in those with Zika; while viral RNA in urine, persisted for longer in patients with dengue. Thus, by day 7 (between days 10 and 17 after symptoms onset) the proportion of patients with either viral RNA in either plasma or urine was close to 10% in both groups (Fig. 2).

**Fig. 3:**
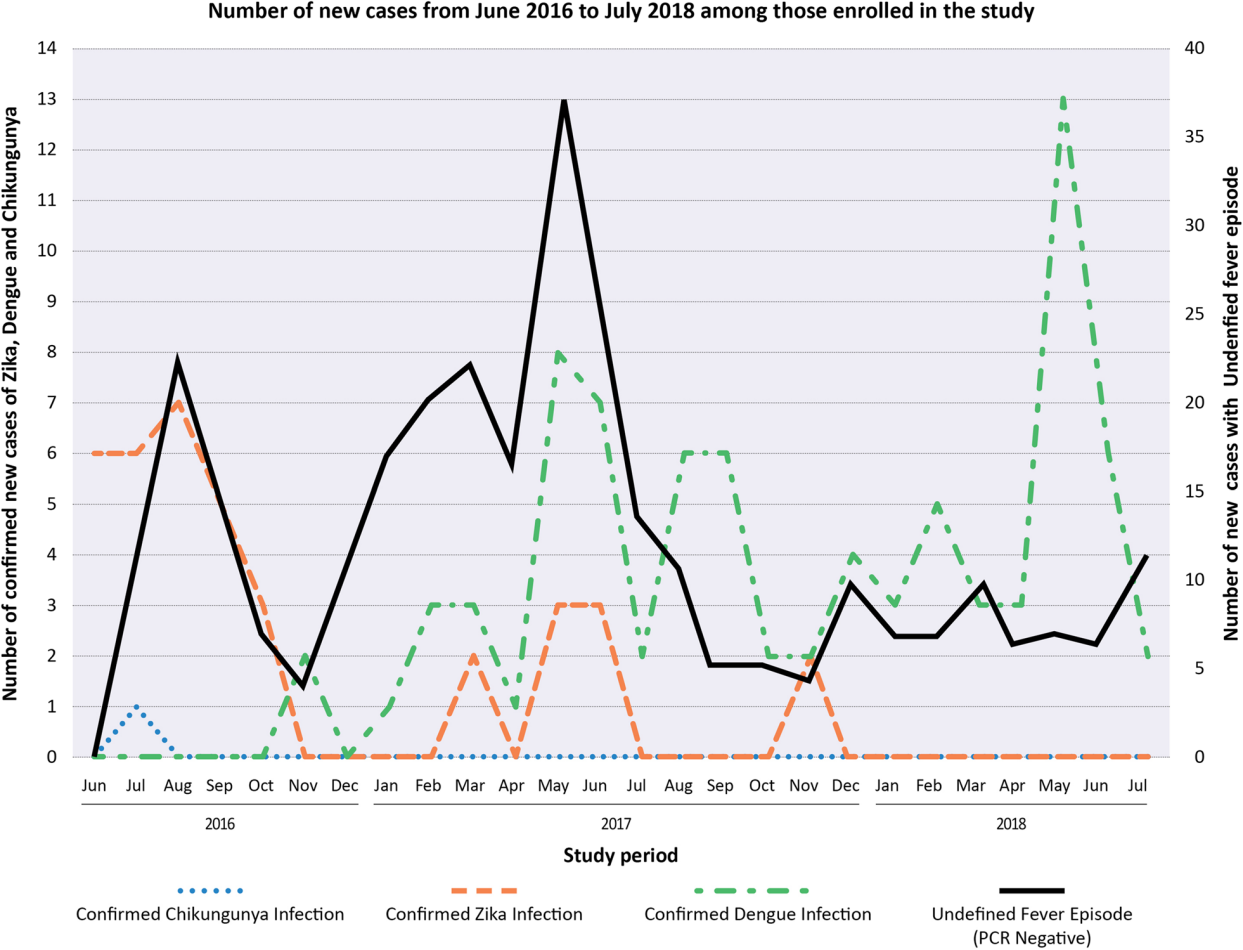
Distribution over time of confirmed cases of Zika, dengue and chikungunya infections, and undefined illness episodes of patients enrolled in the Zik01 cohort between June 2016 and July 2018 in the city of Tapachula, Chiapas in Mexico. Description: Epidemic curves over the 2-year period of enrollment by definitive diagnosis

There was a different annual pattern in the frequency of the occurrence of Zika, dengue and UIE. During the early part of the study (Jun–Dec 2016) Zika and UIE were the most frequently diagnosed illnesses in the cohort, while only one case of Chikungunya and sporadic dengue cases were diagnosed by the end of the year. During 2017, dengue and UIE peaked, particularly during the summer, but occurred throughout the year, with a few cases of Zika diagnosed during the first semester. In contrast, in the latter part of the study (Jan–Jun 2018) we observed no Zika cases, UIE plateaued at a few monthly cases and dengue occurred throughout the period with a large peak during May–June 2018 (Fig. 3).

## Discussion

In this prospective, observational cohort study conducted in the city of Tapachula, Chiapas, we observed that overall over the entire course of the study, UIE was the most frequently observed acute illness in people who met criteria for probable Zika in this area, followed by dengue. However, in the period early after Zika introduction to Mexico (2015–2016) through this border [20], most acute febrile or rash illnesses were attributed to Zika and UIE. Later, the proportion of Zika decreased, and dengue increased proportionally along with UIE; during the third year we observed no Zika, a large seasonal outbreak of dengue occurred, and UIE proportionally decreased. During the whole study period we observed no co-infections as has been reported to occur with relative frequency in dengue endemic areas in Latin American after the emergence of Zika and chikungunya [15, 21–23].

We also observed differences in demographic characteristics and time between symptoms onset and the first medical visit between groups: participants with dengue were more frequently men, considerable younger, and had lower educational levels than people with Zika and UIE. Although Rodriguez-Barraquer et al. did not find any association between age and immunity to Zika virus in Salvador, Brazil during the initial outbreak, there was a clear increasing prevalence with age in dengue seroprevalence [24]. Our results are in agreement with previous studies in Nicaragua, Brazil and Honduras, where patients with Zika tend to be older, less frequently men, or more educated than people with dengue and other febrile episodes [19, 25, 26]. This is consistent with differences in risk-environments in neighborhoods, work, but also determined by mobility, age, gender, individual behavior, education, and socioeconomic status [9, 27, 28]. We also observed that it took longer after symptoms onset for people with dengue to seek care than for patients with Zika and UIE. While this might be expected in a dengue endemic region where people would seek care earlier for atypical or unfamiliar symptoms, we are not aware of other studies having noticed these differences. If this is observation is confirmed, it is worrisome that patients with dengue in the region might be delaying care for a potentially life-threatening disease. Observed differences in sexual behavior, awareness of transmission within neighborhoods and schools at diagnosis, proximity to standing water sources, availability of protective window screens and bed nets suggest that despite common transmission routes and environmental conditions, local differences might determine heterogeneity in exposed groups, at least during Zika introduction. The interacting social and environmental changes in this region have enabled the introduction, rapid expansion and emergence or re-emergence of vector-borne infections [4, 21], but it is difficult to determine distinctive transmission patterns. Here we observed that early during Zika introduction, Zika and dengue tended not to co-circulate locally at the same intensity. We observed that participants with Zika reported recent sexual intercourse with partners with acute febrile-rash illnesses more frequently than people with dengue and UIE (overall and in the participants between 18 and 64 year old). While this is consistent with a relatively frequent sexual transmission of the disease, our study design and methods does not allow us to draw confirmatory conclusions from this observation. In a prospective observational cohort in Puerto Rico during the same period, researchers found evidence that among household contacts of symptomatic patients with Zika, sexual behavior appears to have been an important risk factor for transmission [29]. None of the household cohort participants in our study were linked to symptomatic patients with Zika in the febrile-rash cohort.

We observed different timeframes during which patients presented as test positive for viral RNA in blood and urine, but in general RNA in the urine persisted up to 10 days after symptoms onset in a fifth of patients with dengue, and a up to a tenth of patients with Zika and dengue until 2 weeks after symptoms onset. The latter might be relevant for clinical diagnosis or surveillance purposes. Current WHO and CDC testing guidance recommends using molecular test in bodily fluids (but not urine) during the first 7 days of symptoms onset [30, 31], but our data suggests that potential utility of testing for dengue in urine and plasma beyond the first week.

This study has several limitations. The cohort enrolled participants who presented for clinical care and may not represent the entire spectrum of disease in participants who do not present for care. This cohort may represent a more severe subset of people with disease who choose to present for care. While the purpose of the household cohort was to enroll this population, none of the participants tested positive for Zika or dengue and all of them were linked to participants with UIE. Moreover, our results might be only applicable to patients that met eligibility criteria according to previously established case definitions, but might have been missed clinical manifestations not included in the modified PAHO definition. A different subset of patients with different patterns of clinical manifestations might have been enrolled with a different probable case-definition [19]. We used the identification of viral nucleic acids in clinical samples of participants to define confirmed cases of infection. RT-PCR for nucleic acid detection might have limitations in sensitivity, particularly for patients with Zika infection, [32] which might misclassify some patients with Zika or dengue as UIE. However, the repeated measurement of viral RNA in both blood and urine samples may help minimize this potential source of error [14]. The strength of this study was that participants who presented for clinical care for probable Zika were thoroughly evaluated for various clinical and laboratory aspects of the diseases. The study has a robust design and predefined and standardized procedures, on which we prospectively collected detailed information on clinical and laboratory variables. Also, we enrolled patients in a variety of care centers that encompass all levels of care (primary to tertiary care) and healthcare institutions providing services in the area (state and national, and under different type of funding mechanisms, including services for those uninsured), which allowed to include patients with different backgrounds and heterogeneously distributed within the area.

## Conclusions

In this prospective observational cohort to characterize Zika, dengue, chikungunya during the first 2 years of Zika introduction to a dengue hyper-endemic region, frequency of Zika peaked and fell while dengue progressively increased over a two-year period, ending with dengue predominating in 2018. We also observed different, though widely overlapping, epidemiologic patterns between Zika, dengue and UIE. Our study suggests that, at least in urban settings in tropical areas, microenvironment characteristics might determine heterogeneous exposure (in time and space) to co-circulating pathogens. It also shows that identification of nucleic acids of Zika and dengue for surveillance and diagnostic purposes might be useful if measurements are performed for longer than currently recommended.

## Supplementary Information

**Additional file 1.** STROBE Statement—Checklist of items that should be included in reports of *cohort studies*.

## Data Availability

The datasets used and/or analysed during the current study are available from the corresponding author on reasonable request.

## Abbreviations

DenV: Dengue virus infection
MoCA: Montreal cognitive assessment
RT-PCR: Reverse transcriptase-polymerase chain reaction
UIE: Undefined illness episode
WHODAS 12: World Health Organization Disability Assessment Schedule 2
Zika: Zika virus infection
